# Photoelectron circular dichroism of aqueous-phase alanine†

**DOI:** 10.1039/d5sc00167f

**Published:** 2025-04-07

**Authors:** Dominik Stemer, Stephan Thürmer, Florian Trinter, Uwe Hergenhahn, Michele Pugini, Bruno Credidio, Sebastian Malerz, Iain Wilkinson, Laurent Nahon, Gerard Meijer, Ivan Powis, Bernd Winter

**Affiliations:** a Fritz-Haber-Institut der Max-Planck-Gesellschaft Berlin 14195 Germany dstemer@fhi-berlin.mpg.de winter@fhi-berlin.mpg.de; b Department of Chemistry, Kyoto University Kyoto 606-8502 Japan thuermer@kuchem.kyoto-u.ac.jp; c Institute for Electronic Structure Dynamics, Helmholtz-Zentrum Berlin für Materialien und Energie Berlin 14109 Germany; d Synchrotron SOLEIL St. Aubin 91190 France; e School of Chemistry, The University of Nottingham Nottingham NG7 2RD UK

## Abstract

Amino acids and other small chiral molecules play key roles in biochemistry. However, in order to understand how these molecules behave *in vivo*, it is necessary to study them under aqueous-phase conditions. Photoelectron circular dichroism (PECD) has emerged as an extremely sensitive probe of chiral molecules, but its suitability for application to aqueous solutions had not yet been proven. Here, we report on our PECD measurements of aqueous-phase alanine, the simplest chiral amino acid. We demonstrate that the PECD response of alanine in water is different for each of alanine's carbon atoms, and is sensitive to molecular structure changes (protonation states) related to the solution pH. For C 1s photoionization of alanine's carboxylic acid group, we report PECD of comparable magnitude to that observed in valence-band photoelectron spectroscopy of gas-phase alanine. We identify key differences between PECD experiments from liquids and gases, discuss how PECD may provide information regarding solution-specific phenomena — for example the nature and chirality of the solvation shell surrounding chiral molecules in water — and highlight liquid-phase PECD as a powerful new tool for the study of aqueous-phase chiral molecules of biological relevance.

## Abstract 2

Liquid-jet photoelectron circular dichroism provides a site- and chemical-state selective probe of chiral molecules in solution.

## Graphical abstract

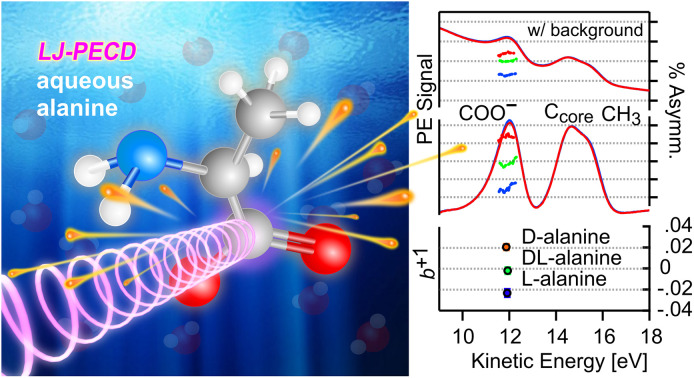

## Introduction

Chirality describes a broad class of objects that share the property of being non-superposible with their mirror images. This general property of chirality may be found at all size scales and levels of complexity in Nature, from human hands and sea shells down to small molecules. Many of the molecules most essential for life on Earth are chiral, including macromolecules such as proteins and DNA as well as smaller building blocks of life such as amino acids and sugars. All chiral biomolecules have been found to be overwhelmingly present in only a single enantiomeric form in living organisms, despite the mirror-image enantiomeric forms of a chiral molecule exhibiting identical physical and chemical properties in achiral environments (exempting small anticipated energetic differences between enantiomers due to parity-violating effects in the weak interactions).^1^ The origins of this asymmetry in biology remain unresolved. In chiral environments, the chemical activity of the different enantiomers of a chiral molecule can differ dramatically. As such, experimental methods capable of distinguishing between enantiomers under biologically relevant conditions are of direct interest for chemical and life sciences. Absorption-based electronic circular dichroism (ECD) describes the asymmetry in absorption of left- or right-handed circularly polarized light (CPL) in the UV-visible range by different enantiomers of a chiral molecule. ECD is widely used as a means to determine the degree of enantiomeric excess for chiral molecules in solution.^2^ However, it is dependent on the interaction between the electric and magnetic dipoles of the chiral system with the CPL, and correspondingly the intrinsic effect magnitudes are small, generally on the order of 0.01%.^3^

In 2001, it was first experimentally demonstrated that one-photon photoionization of chiral molecules by circularly polarized synchrotron radiation may also be used to differentiate between enantiomers.^4^ This photoelectron circular dichroism (PECD) — predicted theoretically 25 years earlier by Ritchie^5,6^ — manifests as a forward–backward asymmetry in the measured photoelectron flux along an experimental axis defined by the direction of light propagation. Arising purely due to electric-dipole interactions, PECD is much more pronounced than ECD, exhibiting asymmetries on the order of 1% up to a few 10's% for randomly oriented molecules.^7^ Alignment of molecules has been demonstrated to further increase PECD effect sizes.^8–10^ PECD appears to be a general feature in photoionization of chiral systems by CPL, having been observed in core-level and valence-band photoemission from terpenes^11–14^ as well as from molecular dimers and larger clusters,^15,16^ amino acids,^17–19^ organometallic complexes,^20^ and nanoparticles.^18^ In addition to the traditional strengths of photoelectron spectroscopy, including chemical-state and site specificity, PECD is uniquely sensitive to small differences in molecular electronic structure and is capable of clearly distinguishing between different structural and conformational isomers of a chiral molecule.^21,22^ PECD is a powerful method capable of much more than the resolution of enantiomers and the determination of enantiomeric excess. These very appealing analytical capabilities have driven the growing field of laser-based multi-photon PECD since 2012.^23–25^

Water is critically important for biochemistry, playing an active role in determining the functionality of amino acids, proteins, and DNA through structure stabilization and mediation of intra- and intermolecular interactions.^26^ For chiral molecules in solution, interactions with solvent molecules, even achiral solvents such as water, may profoundly influence both the magnitude and the sign of measured chiroptical effects.^27^ Additionally, it is possible that achiral solvent molecules may themselves adopt chiral arrangements within the first solvation shell around a chiral solute, mimicking to some extent the solute's chiral structure.^28^ Understanding the complex interactions between chiral solutes and achiral solvents is clearly a necessary prerequisite for developing deeper insight into the chemical activity of chiral molecules in solution, whether in biochemical or synthetic contexts. Core-level PECD seems uniquely suited for an investigation into the remarkably active role of solvent molecules in determining chiral solute properties. However, PECD of liquid-phase samples has only very recently become experimentally feasible.^29,30^

The development and growth of liquid-jet photoelectron spectroscopy (LJ-PES) over the past few decades has led to the solution of many of the technical obstacles that long prohibited the study of volatile liquids under the vacuum conditions required for PES.^31,32^ However, the application of PECD to liquids, and particularly to aqueous solutions, introduces a number of new experimental challenges that are not present for gas-phase studies. PECD, based upon the scattering and interference of departing partial electron waves, exhibits a strong kinetic-energy dependence, with the most pronounced effects manifesting for photoelectrons with kinetic energies of less than approximately 15 eV.^11^ For PES of condensed-phase systems this poses a clear challenge due to the unavoidable convolution of primary photoelectron spectral features in this kinetic-energy range with the high-intensity low-energy electron background composed of electrons inelastically scattered in the sample bulk. For the cases of liquid water and of bulk-soluble species in aqueous solution, the opening of efficient quasi-elastic scattering channels within the same kinetic-energy range additionally leads to the broadening of photoelectron features and the homogenization of photoelectron angular distributions.^33,34^

Despite these challenges, we have previously demonstrated the viability of liquid-phase PECD measurements for the chiral molecule fenchone as a neat liquid.^30^ We now report PECD in core-level photoionization of aqueous-phase alanine — the simplest chiral amino acid — at different solution pH values corresponding to the cationic, zwitterionic, and anionic form of the molecule. We summarize the results of our measurements, highlight the key differences in measuring PECD in the aqueous and gas phases, and discuss the potential influence of water solvation on PECD. We outline new opportunities for aqueous-phase PECD going forward, including the relatively straightforward application of PECD to study cationic or anionic chiral molecules; a topic that has only recently begun to be explored.^35–37^ More generally, this study constitutes the first core-level PECD investigation of chiral amino acids in any context. Liquid-phase PECD studies of the elementary constituents of life are highly relevant to *in vivo* biological conditions. We present a first step toward a bottom-up approach to understanding biomolecular complexity through the preparation of simple aqueous solutions, enabling the study of fragile thermolabile chiral species such as amino acids, peptides, sugars, and nucleic acids, whose vaporization into the gas phase with sufficient density to enable core-level PES is challenging.

## Experimental considerations

**Fig. 1:**
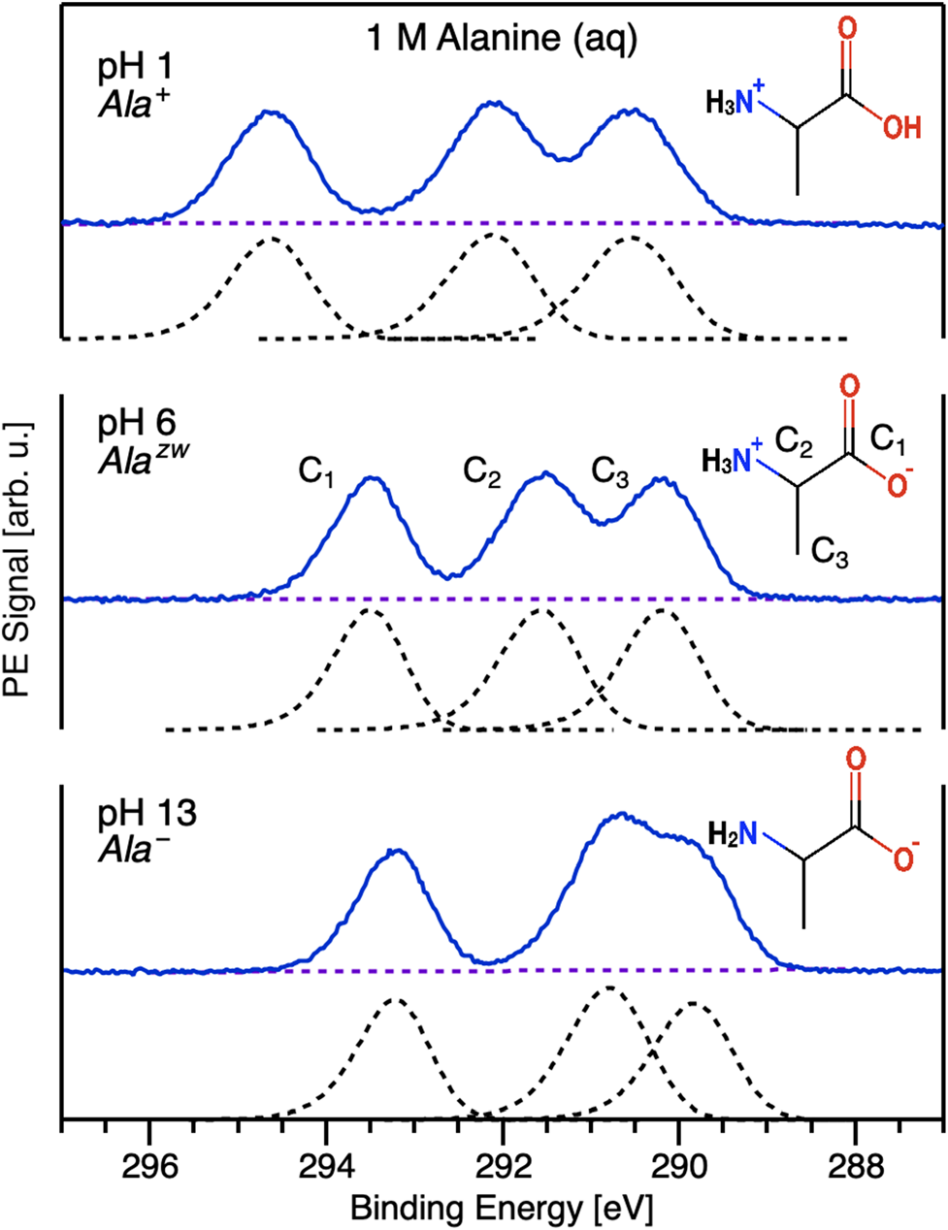
Representative C 1s photoelectron spectra corresponding to aqueous-phase alanine's three chemically distinct carbons measured with *hν* = 480.46 eV photons under acidic (top), neutral (middle), and basic (bottom) aqueous conditions. Measurements were conducted with 1 M aqueous solutions of l-alanine. The dashed black curves are fits to the data following the subtraction of background signals (dashed purple lines) using exponentially modified Gaussian (EMG) profiles.^38^ The EMG asymmetry parameter was constrained to be between 0.2 and 0.3, and these values were kept constant in fits across different pH conditions. The ratios of peak widths were also constrained to differ by no more than 5% across the three fits. Molecular structures of the dominant protonation state of alanine at the various pH values investigated are shown as insets.

In this work, we focus on core-level C 1s measurements of aqueous-phase alanine. Alanine has three chemically distinct carbon atoms — a carboxylic-acid group, a chiral central carbon adjacent to an amine group, and a methyl group, which we denote C_1_, C_2_, and C_3_, respectively — resulting in a solution-phase C 1s PE spectrum with three distinct features that overlap to varying degrees depending on the solution pH/alanine protonation state (Fig. 1).

To track chemical shifts upon protonation/deprotonation of alanine's functional groups, we measured C 1s PE spectra for aqueous solutions of alanine with solution pH values of 1, 6, and 13 using 480.46 eV photons. These pH values were chosen based on the p*K*_a_ values of alanine's functional groups (2.3 and 9.9 for alanine's carboxylic acid and amine groups in water, respectively) and ensured that, for a given solution pH, >95% (>99% for pH 6 and 13) of the alanine molecules in solution adopted a single charge state. For these measurements, we employed the energy-referencing scheme that we have developed over the past years.^39–41^ Briefly, this method enabled us to correct for the influence of electrokinetic charging and other stray fields by applying a suitable bias voltage to the liquid jet prior to measurement, thereby increasing the kinetic energy (KE) of all photoelectrons commensurately with the bias applied and revealing the secondary electron cutoff of the spectrum, which represents a true zero kinetic-energy reference for condensed-phase experiments. We note that it was not practical to employ these measures for the low-kinetic-energy PECD measurements discussed later, and thus the axes displayed in subsequent figures represent as-measured kinetic-energy values, which may be subject to inaccuracies due to spectrometer offsets and the presence of streaming potentials and other stray fields.^42,43^

Under strongly acidic conditions, alanine is predominantly in a cationic state (Ala^+^), with the amine functional group protonated (Fig. 1, top). In this state, despite some overlap between C_2_ and C_3_, the three carbon PE features are easily resolvable owing to pronounced chemical shifts arising due to the different chemical environments of the carbon sites. The highest binding energy (BE) feature corresponds to photoelectrons originating from the relatively electronegative COOH group (C_1_). The central PE feature corresponds to the chiral center adjacent to the amine group (C_2_), and the final feature is attributable to the methyl group (C_3_). These assignments are consistent with previous solid-state and gas-phase work.^44,45^ As can be seen by comparing the pH 1 and pH 6 data in Fig. 1, deprotonation of the carboxylic acid for zwitterionic Ala^zw^ is accompanied by a noticeable chemical shift of the C_1_ peak of approximately 1.12 eV toward lower BE, reflecting the increasing electron density at that site upon deprotonation. C_2_ and C_3_ also exhibit shifts to lower BE, albeit of smaller magnitudes, indicating their relatively lower sensitivity to the changing electron density on the neighboring functional group. This is likely a consequence of effective charge screening by nearby water molecules.^46^ At this pH, all three primary PE features are still readily resolvable. At pH 13, alanine is found in the anionic form, Ala^−^, with the carboxylic acid deprotonated and the amine group neutral. In this case, we observe a chemical shift of 0.77 eV toward lower BE for the C_2_ peak, corresponding to an increase in electron density at that group compared to the pH 6 case. This chemical shift results in substantial overlap of the C_2_ and C_3_ peaks. We note that the C_1_ peak remains clearly isolated from the C_2_ and C_3_ features for all charge states, thereby simplifying the determination of PECD for this carbon site. The binding energies for C_1_, C_2_, and C_3_ for solution pH 1, 6, and 13 are summarized in Table 1.

**Table 1:** C 1s binding energies (BEs) for each of alanine's carbon groups as a function of solution pH

Solution pH	BE C1 [eV]	BE C2 [eV]	BE C3 [eV]
1	294.64	292.12	290.53
6	293.52	291.56	290.19
13	293.25	290.79	289.81

By comparing the relative peak area of C_1_, C_2_, and C_3_, we can draw initial inferences about the orientation of surface-localized molecules. Normalizing to the area of C_1_, we find that the C_1_ : C_2_ : C_3_ ratios are 1 : 1.09 : 1.13, 1 : 1.12 : 1.09, and 1 : 1.23 : 1.07 for Ala^+^, Ala^zw^, and Ala^−^, respectively. Assuming that the C 1s photoionization cross section differs only negligibly for C_1_, C_2_, and C_3_, a completely random molecular orientation distribution would result in a peak-area ratio of 1 : 1 : 1. A deviation from this ratio suggests partial molecular orientation for molecules near the liquid–vapor interface, with lower peak areas indicating that the photoionized groups are located deeper within the solution bulk.^47^ Our results suggest that C_1_ appears to reside further in the solution bulk than C_2_ or C_3_ at all solution pH values probed. The disparity in the C_1_ : C_2_ ratio is smallest for Ala^+^ and most significant for Ala^−^, where the charged carboxylic group is likely to be fully solvated. However, due to its overall hydrophilicity, alanine does not exhibit strong surface propensity,^48^ and oriented molecules near the liquid–vapor interface are likely to make up only a fraction of the total molecules probed. Nevertheless, these potential effects are important to keep in mind, particularly for PECD measurements, which inevitably involve low-kinetic-energy electrons and are thus particularly surface sensitive.

Similarly to our previous study of PECD from neat liquid fenchone,^30^ we determined PECD using synchrotron radiation and an in-vacuum liquid-jet photoemission spectrometer *via* sequential measurements of photoelectron flux upon photoionization of aqueous alanine solutions with left- and right-handed CPL (see Experimental section below for additional details). The implemented beamline settings corresponding to each type of polarization were confirmed previously through measurements of PECD from gaseous fenchone.^29,30^ Electrons were detected in the backward direction, with our electron analyzer mounted at ≈50° with respect to the axis of light propagation, approximating a magic-angle geometry of 54.74°.^29^ This angle was chosen due to technical considerations of compatibility with the open port at the P04 soft X-ray beamline, where these measurements were performed.

For single-photon photoionization of a randomly oriented sample,^49^ the normalized photoelectron angular distribution (PAD) within the electric-dipole approximation is given by:

1 *I*^p^(*θ*) = 1 + *b*^p^_1_*P*_1_(cos *θ*) + *b*^p^_2_*P*_2_(cos *θ*)

here, *I*^p^(*θ*) is the photoionization intensity at an angle *θ* measured with respect to the photon-propagation direction for a given light polarization (*p* = ±1 for circularly polarized light and 0 for linearly polarized light, respectively). *P*_*n*_ represents the Legendre polynomial of the *n*th order. The two coefficients *b*^p^_1_ and *b*^p^_2_ completely encompass the target-specific contribution to the PAD under a fixed set of experimental conditions. For achiral molecules, *b*^p^_1_ = 0 and thus the PAD is described by *b*^p^_2_, more commonly denoted *β* for experiments involving linearly polarized light (*i.e. b*^0^_2_ = *β*, while *b*^±1^_2_ = −*β*/2).^50^ For chiral molecules, *b*^±1^_1_ may be non-zero and *b*^+1^_1_ = −*b*^−1^_1_, changing sign upon inversion of the handedness of CPL (or of the enantiomer). An asymmetry factor, *G*, may be defined as the simple difference between left- and right-handed PE intensities divided by their average. Using eqn (1) and noting that *P*_1_(*x*) = *x*, we then have:2
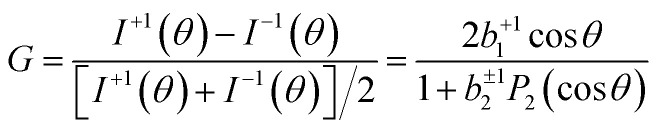


It can be seen that this provides a means to extract *b*^+1^_1_ although, in general, a knowledge of *b*^±1^_2_ is also necessary. However, *P*_2_(cos(54.74°)) = 0. Thus, at magic angle:3
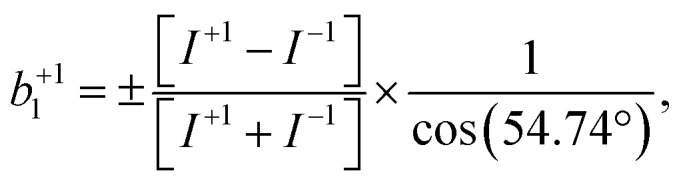
the negative sign applying when, as here, measurement is made in the backwards hemisphere. We note that *P*_2_(cos(50°)) ≠ 0, and we discuss the relevant consequences of our near magic-angle measurement geometry in greater detail below.

As *b*^+1^_1_ simply changes sign upon exchange of enantiomer, in principle only a single pair of measurements for a single enantiomer with left- and right-handed CPL is necessary for its determination at any given energy using eqn (2). The normalized intensities *I*^p^(*θ*) are obtained from the experimental photoelectron intensities at a specified electron energy and detection angle *θ*, but require correction accounting for possible variation in photon flux and other sources of medium term experimental drift. Despite our best efforts to ensure that the fluxes of left- and right-handed CPL were equal – monitored *via* a photodiode – small persistent differences in light intensity of 1–3% remained and so data normalization was essential. In gas-phase PECD studies, 4π imaging spectrometers enable collection of the total ionization yield, and thus enable direct normalization.^12^ In the present experimental arrangement, the total ionization yield cannot be monitored and an alternative intensity-correction procedure utilizing the baseline signal was adopted. It is assumed that the background of scattered electrons is achiral and essentially independent of light polarization state. We therefore scaled all left- and right-handed CPL data (hereafter denoted as + and − spectra) to ensure equal intensity of the spectra at the low- and high-KE edges, where only an achiral background is present.

**Fig. 2:**
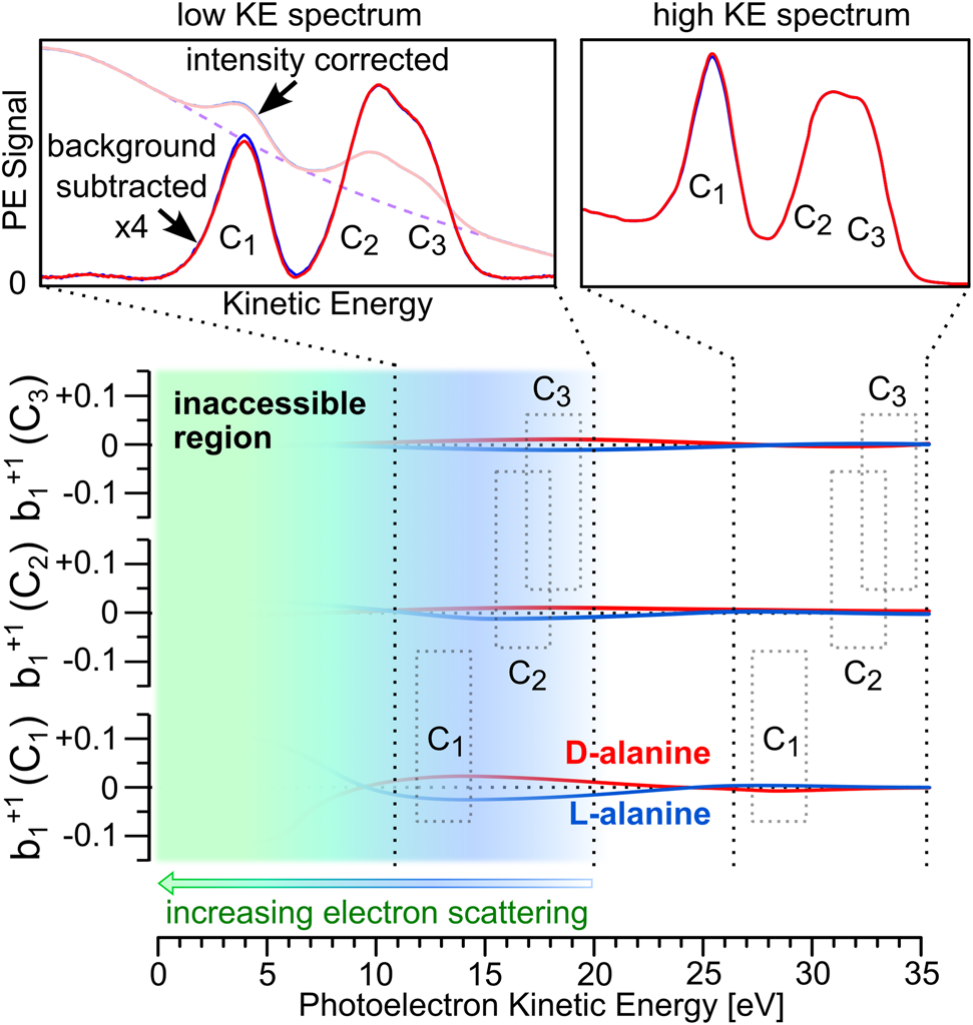
(Top) Illustrative aqueous-phase (pH 13) alanine C 1s photoelectron spectra corresponding to right- and left-handed circularly polarized light (red and blue lines, respectively). The high-energy spectra (right) exhibit low background signal, but vanishing photoelectron circular dichroism (PECD). The low-energy spectra sit atop a large background signal, but reveal significant PECD following subtraction of background signal (purple lines). (Bottom) Hypothetical *b*^+1^_1_ parameters for each of alanine's carbon groups as a function of peak kinetic energy. The blue shaded zone represents the onset of significant kinetic-energy-dependent elastic or quasi-elastic electron scattering. Within the green region, scattering of primary photoelectrons is sufficient to make resolution of the photoelectron features unfeasible. Values of *b*^+1^_1_ are currently inaccessible for this system within this kinetic-energy range. Dashed gray boxes represent approximate peak positions for the spectra shown above. The carbon atoms C_1_, C_2_, and C_3_ are identified in Fig. 1.

It is also vital that the baseline is accurately modeled such that the background beneath the peaks of interest can be reliably removed from the intensities *I* used to determine *b*^+1^_1_. It can be seen from eqn (2) that while any residual background present in the suitably scaled *I*^+^ and *I*^−^ will self-cancel in the numerator, this does not apply to the denominator, [*I*^+^ + *I*^−^], whose magnitude would hence be overestimated and lead to an erroneous underestimation of the derived |*b*^+1^_1_|. This challenge is exacerbated for experiments with dilute condensed-phase chiral molecules where the signal-to-background ratio is much reduced (see Fig. 2, top). Details regarding the background-subtraction method employed may be found in the ESI.†

At the near-magic-angle geometry of *θ* = 50° (in the backward direction) adopted for these experiments, the polynomial *P*_2_(cos *θ*) ≠ 0 and some residual influence of the *b*^±1^_2_ parameter is unavoidably included in the measurement (see eqn (2)). However, there are currently no published values of *b*^±1^_2_ for core-level photoionization of alanine. To obtain an estimate of the significance of the remnant *b*^±1^_2_ contribution, we calculated the value of *b*^0^_2_ (*β*) for low-energy (KE = 0–25 eV) photoelectrons generated upon C 1s photoionization of gas-phase Ala^0^ (details may be found in the ESI, see Fig. S3†). We found that for photoionization of C_1_, the absolute value of *β* is less than 0.8 within this entire KE range, decreasing to less than 0.5 for photoelectrons with KE < 15 eV. Although aqueous-phase alanine will undoubtedly yield different values of *β* due to differences in conformation and the charge state of its functional groups, this calculation nevertheless provides a rough estimate. As a comparison, the measured value of *β* for O 1s photoionization of gas-phase water at 25 eV KE is approximately 1.6, whereas for liquid water at the same energy it is 0.6.^34^ For both environments, *β* trends strongly toward 0 for lower photoelectron kinetic energies. This decrease in *β* as measured from liquid *versus* gaseous water was attributed primarily to highly efficient elastic or quasi-elastic^33^ scattering of photoelectrons by water molecules in the liquid. As water remains by far the likeliest scatterer of photoelectrons for the 1 M aqueous solutions of alanine studied in this work, it seems reasonable to expect a similar degree of PAD isotropization for *β* as measured in C 1s photoionization of these solutions. Conservatively assuming a *β* value of 0.5 as an upper limit for aqueous-phase alanine and using eqn (2) while recalling that *b*^±1^_2_ = −*β*/2 and simply averaging over the acceptance angle of the analyzer (≈±15°),^29^ the effect would be to scale our measured values of *b*^+1^_1_ by a factor of 0.97. Such a change does not alter the results of this study, and we do not consider it further in subsequent discussion.

For a given aqueous alanine solution (enantiomer, pH), we conducted C 1s LJ-PES measurements as a function of photon energy in the range of 302–310 eV, resulting in C_1_ peaks in the KE range of 9–17 eV. For each photon energy, we measured 6–10 PE spectra before changing the polarization of the light, resulting in a measurement time of roughly 30–60 minutes per sample. We compared repeats using the same handedness of CPL for a given photon energy over time to ensure that our experimental conditions were reproducible during the measurement period.

In practice, and particularly due to the pioneering nature of this study, it is advantageous to determine *b*^±1^_1_ for both enantiomers in order to detect any influence of instrumental and experimental asymmetry. Confirming the anticipated enantiomeric mirroring of the *b*^+1^_1_ parameter assures the molecular origin of the observed asymmetry. Wherever possible, we also made the same observations for solutions of racemic dl-alanine, for which no asymmetry is expected. Due to the time-intensive nature of the experiments, which spanned multiple months of synchrotron measurements across four years, it was necessary to find a balance between the photoelectron KE range and enantiomer and protonation state of alanine investigated. This balance is reflected in the greater number of experimental data points measured for low-KE photoelectrons and for Ala^−^ (see for reference figures Fig. S4, S8, and S12†).

## Results

**Fig. 3:**
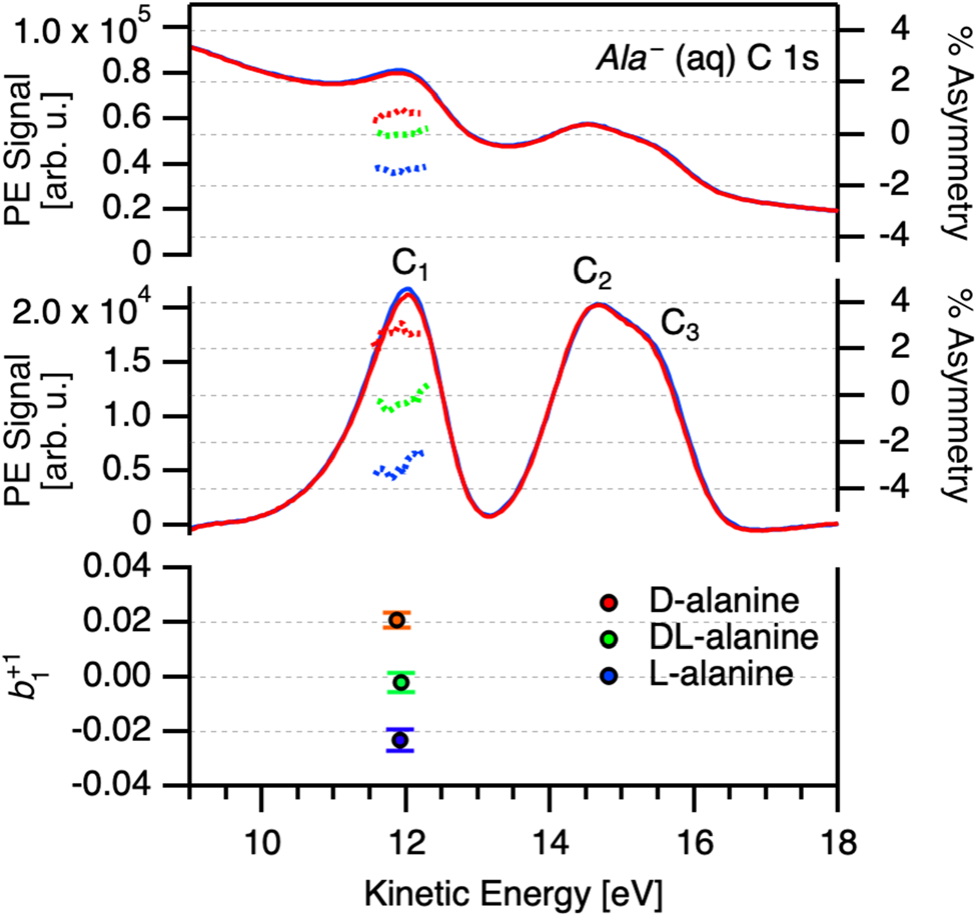
(Top) Representative intensity-scaled C 1s photoelectron spectra of 1 M l-alanine aqueous solution (pH 13) measured at *hν* = 305 eV for left- and right-handed circularly polarized light (blue and red curves, respectively), along with the calculated percent difference between the spectra for C_1_ group prior to background subtraction (right axis). (Middle) Background-subtracted data, along with calculated percent difference between the spectra for C_1_ (right axis). (Bottom) Values of *b*^+1^_1_ obtained through the same process for d-, l-, and racemic dl-alanine (red, blue, and green points, respectively). The error bars of these points represent the standard deviation of the percent-difference data shown in the middle panel. The spectra shown here are averages of ten acquisitions, as is typical for the data discussed in this study.

**Fig. 4:**
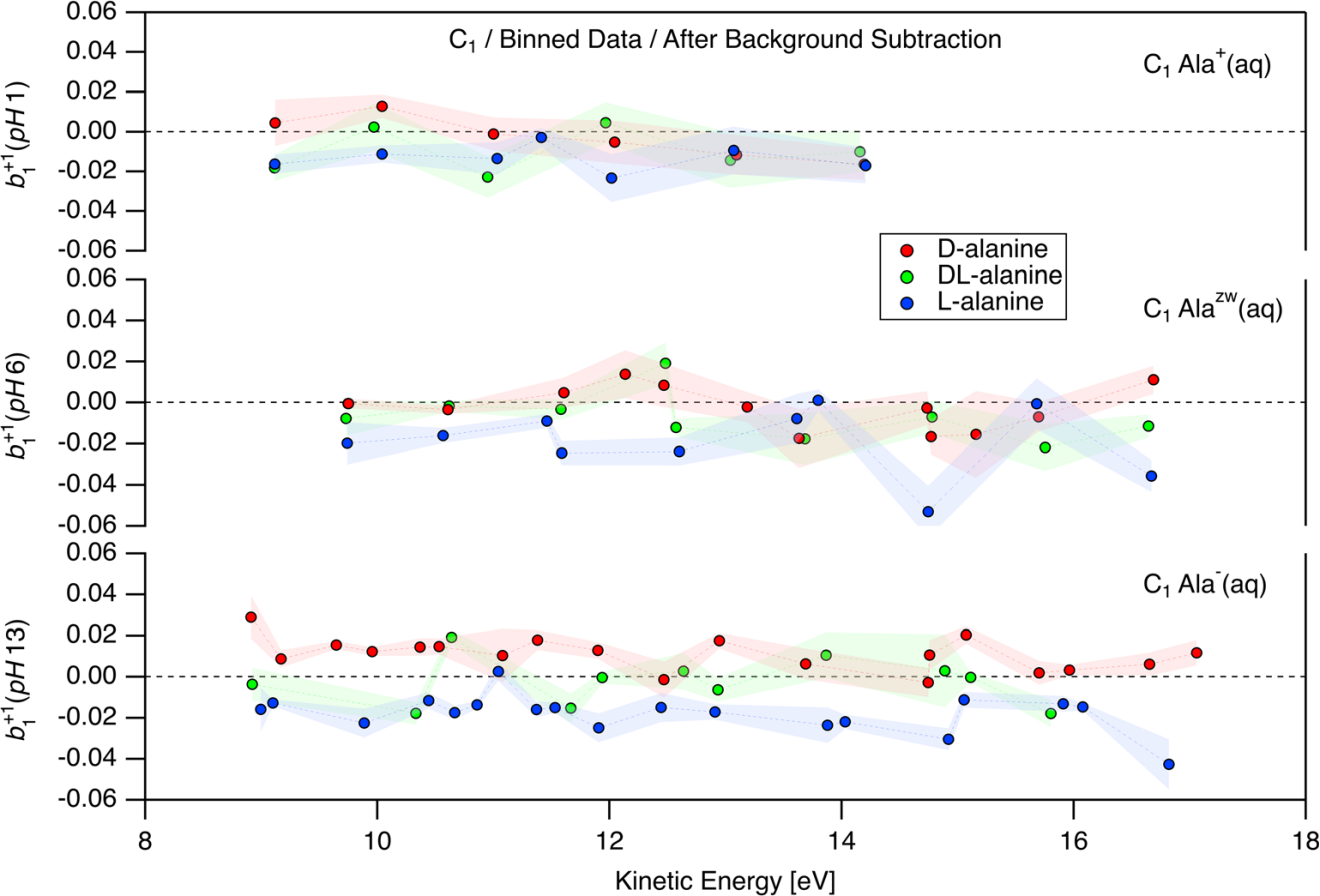
Values of the *b*^+1^_1_ photoionization parameter obtained for C 1s measurements of aqueous solutions of d-, l-, and dl-alanine (red, blue, and green points, respectively) at pH 1, 6, and 13 (top, middle, and bottom; corresponding to the cationic, zwitterionic, and anionic form of the molecule, respectively). All *b*^+1^_1_ values shown correspond to photoionization of the C_1_ carboxylic group. The data displayed is the result of binning the results of individual measurement sets using a kinetic-energy window of 250 meV. The shaded error bars represent the combined error of the binned data points. A description of the error propagation may be found in the ESI.†

Following the process outlined in the ESI† and using eqn (2), we determined a single value of *b*^+1^_1_ per carbon center for each pair of + and − measurements for a given enantiomer, solution pH, and photon energy (Fig. 3). The data were then averaged within 250 meV KE windows for visualization, and to provide a realistic accuracy estimate for our measured photoelectron kinetic energies (see above for factors influencing the accuracy of “as-measured” kinetic energies). The complete datasets without averaging are presented in the ESI (Fig. S5, S9, and S13†). The analysis was carried out blind with respect to the enantiomer, such that we analyzed all the data without knowing whether any given pair of measurements corresponded to a solution of l-, d-, or dl-alanine. An overview of the *b*^+1^_1_ parameters obtained for C_1_ at different pH values is presented in Fig. 4. Beginning with the pH 1 data, corresponding to Ala^+^, weak enantiomer-dependent asymmetry in *b*^+1^_1_ may be seen for photoelectrons with KE = 9 eV. At this energy, l-alanine exhibit a negative value of *b*^+1^_1_, while d-alanine yields a somewhat positive value. These values appear to rapidly trend toward zero at higher kinetic energies, and no significant PECD effect is found above 10 eV. For Ala^zw^ at pH 6, the situation is different. Here, *b*^+1^_1_ is close to zero in the KE range of 9–11 eV, but appears to increase in magnitude briefly around 12–13 eV. From 14–16 eV, the data is noisy and does not indicate clear PECD. At 17 eV, *b*^+1^_1_ increases once more. The sign of *b*^+1^_1_ is the same for the corresponding enantiomer at pH 1. The clearest PECD can be seen for the case of Ala^−^. For this protonation state, *b*^+1^_1_ as measured for C_1_ is clearly non-zero for the entire KE range measured, even up to 17 eV. The sign of *b*^+1^_1_ remains constant throughout the entire range, and is consistent with that observed for Ala^zw^ and Ala^+^. Due to the very time-consuming nature of the experiments, it was not always possible to perform the same number of repeat measurements for d-, l-, and racemic alanine solutions. As such, it is critical to keep in mind that the points plotted in Fig. 4 do not represent the same amount of measurements. This may be seen clearly for the unbinned data shown in Fig. S5.†

We were unable to conclusively identify signatures of PECD for the C_2_ and C_3_ atoms, with the possible exception of Ala^zw^ in the KE range of 15–19 eV (Fig. S8 and S9†). However, due to the limited quantity of data in this KE range for Ala^zw^ and the overlapping nature of these peaks, we do not consider the C_2_ and C_3_ data to convincingly demonstrate PECD. As mentioned above, we dedicated the most time to collecting data points for Ala^−^, which appeared from the onset to yield the most clear PECD. As such, we are the most confident in our results for Ala^−^. It may seem surprising that C_1_ exhibits clear PECD, while C_2_ and C_3_ do not. However, significant site-dependent PECD has previously been reported in C 1s studies of fenchone and glycidol,^14,51,52^ thereby highlighting the sensitivity of PECD to the specific atomic site probed. The datasets corresponding to Fig. 4 for C_2_ and C_3_ are available in the ESI (see Fig. S8 and S12†).

## Discussion

These results provide the only currently available report of PECD in aqueous-phase systems. They reveal a significant PECD effect for aqueous-phase alanine in photoionization of the C_1_ carbon group which falls within the range 0.5–40% commonly encountered in gas-phase PECD studies. The sign of the chiral asymmetry parameter, *b*^+1^_1,_ is consistent across all three protonation states but there are differences in magnitude, with the most pronounced effects seen for the anionic form Ala^−^. Unlike many gas-phase PECD studies, we do not note a significant variation of *b*^+1^_1_ with photon energy.

Any PECD from the C_2_ and C_3_ photoemission essentially must fall below our presently achieved detection limits using a cylindrical liquid jet. Based upon previous experience with this arrangement we can estimate a possible reduction of any photoelectron anisotropy due to electron scattering within the bulk aqueous phase. In liquid water, the angular anisotropy factor (*β*) of the O 1s PAD is reduced by 60% compared to that of gaseous water at KE = 25 eV.^34^ Similarly, in our previous core-level PECD study of liquid fenchone,^30^ we reported a factor of 5 decrease for *b*^+1^_1_ compared to that measured for gas-phase fenchone within the KE range of 9–13 eV.^14^ Hence, we tentatively estimate that our present results for *b*^+1^_1_ may be attenuated by a factor of 3–5. For the C_1_ data shown in Fig. 4, this means that our measured values of *b*^+1^_1_ ≈ 0.02 may correspond to initial *b*^+1^_1_ values of approximately 0.06–0.1 prior to deleterious photoelectron scattering.

There is, *a priori*, no particular surprise that the C_1_ PECD should exceed that of the asymmetrically substituted C_2_ site – the so-called chiral center. The specific details, signs, and magnitudes of PECD are the result of extensive interference between the many continuum partial electron waves during photoionization, making any intuitive prediction or interpretation of signs and magnitudes extremely challenging. It is also well established that PECD asymmetry results from multiple scattering off the spatially extended chiral molecular framework^11,14^ and so is not solely contributed by a designated “chiral center”. Indeed, strong PECD can be observed in molecules with no asymmetrically substituted centers but that display other chiral structural forms such as axial- or propeller-ligand symmetries.^20,53^ Calculations for *e.g.* fenchone^51^ clearly show how the variation in *b*^+1^_1_ values over an extended kinetic-energy range means that the relative signs and magnitudes observed from individual emitter sites will vary with observation energy.

PECD of gas-phase alanine has been reported in the valence region^17,54^ but no C 1s measurements have been published so far. Were they available, simple comparisons would nevertheless be impeded by the different forms (ionic, zwitterionic) that alanine adopts in solution, and that we specifically targeted in this work. Then, even if these charge differences could be incorporated into current modeling, there is in general little value in comparing gas-phase measurements with those made in aqueous media without some understanding of further ways in which solvation may influence the observed PECD. Since our work aims to stimulate the development of theoretical modeling for liquid-phase PECD and, conversely, to show the extensive detail that may then be revealed with the benefit of comprehensive interpretive tools, we next set out further considerations of solvation effects.

The scattering of emitted photoelectrons by bulk water has been alluded to above, but more intimate interactions between a molecule and its solvation shell require consideration. The PECD response is known to be very sensitive to molecular conformation, with great variation in magnitude and sign of the chiral asymmetry even with small changes such as the orientation of a terminal –OH rotamer.^52,55^ This can be a hindrance or a benefit depending on one's perspective and purpose. Solvent–molecule interactions may change the molecular conformational landscape. Approximate treatments for solvation effects have been implemented, and are being widely applied, in many electronic-structure calculation packages. For example, the solvation shell of zwitterionic alanine and its consequent conformational rearrangement have been studied using such methods,^56^ but while these approaches may help identify preferred new conformations in a polar medium, they will lack explicit treatments of individual solvent molecules and their more specific interactions that can provide the level of insight likely to be required for accurate liquid-phase PECD calculations.

A study of microsolvated structures of protonated alanine^57^ details how the addition of solvent molecules leads to the disruption of the intermolecular hydrogen bonds that stabilize and determine gas-phase conformers, replacing them with intramolecular molecule-solvent bonding until the first solvation shell completes with four attached water molecules. Such rearrangement, combined with stabilization of charge in the polar medium, can give rise to the phenomenon of conformational locking in solution, reducing the available conformational space as was inferred for the amino acid proline.^46^ On the other hand, conformational unlocking going from the gas phase to solution has been reported for asparagine,^58,59^ and molecular dynamics simulations of zwitterionic alanine in small water droplets have indicated that alanine does not adopt a single preferred conformer in aqueous solution.^60^ Either an increase or a decrease in conformation flexibility could result in significant PECD changes, and would hence need careful identification. Even if a molecule were conformationally rigid, and so the chiral nuclear scattering potential relatively unaffected by solvation, there is evidence that PECD may be sensitive to the polarization of the electron density by H-bonding,^16^ and could thereby change when H-bonding was disrupted by solvation.

Our results indicate that, even for molecules without significant surface activity, surface-orientation effects may not be trivial. Although it is currently not possible to correlate orientation of molecules at the liquid–vapor interface to observed PECD, given that orientation appears to increase the magnitude of PECD in gas-phase experiments,^8–10^ explicit considerations of both surface propensity and molecular orientation seem to be necessary prerequisites for attempting to model solution-phase PECD.

The central importance of multiple scattering off the chiral molecular potential is particularly evident in core-level PECD when photemission occurs from an essentially spherical – hence achiral – 1s orbital. It is also commonly evident that such chiral final-state scattering may be a long-range effect.^61^ A number of investigations have examined PECD from weakly bound homochiral gas-phase clusters, of species as large as camphor dimers,^15^ that show changes in chiral angular distribution compared to the monomer. In a study of H-bonded *n*-mers of glycidol, a total sign inversion of the strong monomer PECD was observed in the clustered species, with less pronounced differences between the individual *n* = 2, 3, 4 clusters.^16^ More recently, a study of an achiral–chiral complex of phenol-methyloxirane^62^ has shown evidence, namely photoelectron chiral asymmetry from ionization of the phenol chromophore, of induced chirality through such loose bonding. Such chirality transfer between solute and achiral solvent is well documented in, for example, vibrational circular dichroism (VCD).^63–66^ Hence, it seems inevitable that the detailed structure and interactions of at least the first solvation shell^67,68^ would need to be incorporated into a realistic model of aqueous-phase PECD. In general, it appears likely that any effective model of PECD for solvated species must begin with a highly accurate representation of molecular conformation, hydrogen-bonding configuration, and molecular surface propensity and orientation.

One restriction on the currently achieved experimental sensitivity in this investigation stems from the lengthy data acquisition times per measurement and the consequent limitations on the statistical data quality. This is largely a consequence of the detection arrangement, whereby the electron analysis is performed by a hemispherical analyzer that can only measure at a small fixed solid angle and has to be scanned over the electron energy range of interest. Gas-phase PECD measurements were greatly advanced by the early adoption^12^ of velocity map imaging (VMI), which permits energy-resolved photoelectron angular distributions to be detected in a single measurement.^69^ The development of liquid-jet-compatible velocity map imaging is now an ongoing project, which we anticipate will dramatically decrease the amount of time needed for liquid-jet PECD measurements in the future, and thus significantly improve the general suitability of the technique for probing solution-phase molecular chirality.

## Conclusions

We have measured core-level photoelectron circular dichroism (PECD) for aqueous-phase alanine, the smallest chiral amino acid, and examined each of its three charge states (cationic, zwitterionic, and anionic) by the variation of solution pH. A significant PECD was detected for photoionization of alanine's carboxylic-acid functional group. This exhibited a clear magnitude dependence across pH conditions, but did not exhibit a strong kinetic-energy dependence. On the other hand, neither the photoionization of alanine's asymmetrically substituted central carbon, nor its methyl carbon, displayed significant PECD, indicating that the effects for those groups are weaker and below our currently modest experimental sensitivity.

For photoionization of the carboxylic C 1s, PECD is the most pronounced under basic solution conditions, where alanine adopts the anionic form, but is very weak for both the zwitterionic and cationic forms. The differences observed across protonation states may be due to a number of factors, including changes in molecular charge state, conformation, surface propensity, molecular orientation, functional-group-dependent solute–solvent interactions, and first solvation-shell structure.

Theoretical approaches to PECD are currently limited to small gas-phase systems and we identify some of the factors that may be required to develop realistically complete theoretical models for PECD effects in solution. Conversely, this discussion allows one to identify the scope of additional insight into the solvated state that could be exposed by the interpretation of PECD measurements. Experimentally, feasible technique developments of VMI to provide energy- and angle-multiplexed detection, coupled with the introduction of ultrathin liquid-jet technologies^70^ makes clear the potential for significantly improved data collection rates and enhanced detection sensitivity.

Our demonstration of aqueous-phase PECD marks a significant advance in the field of liquid-jet photoelectron spectroscopy. Given its chemical-state and site specificity and enantiomeric sensitivity, liquid-phase PECD is likely to become a remarkably useful analytical method for studying chiral molecules under biologically relevant aqueous conditions.

## Experimental

We carried out all aqueous-phase PECD experiments using our custom LJ-PES apparatus – Electronic Structure from Aqueous Solutions and Interfaces (EASI) – developed specifically for this purpose.^29^ We introduced alanine aqueous solutions into the experimental chamber using a custom quartz capillary, with an inner diameter on the order of 20–30 μm. Solutions were driven by a high-performance liquid chromatography pump (Shimadzu LC-20AD) at a flow rate of 0.8–1.5 mL min^−1^, resulting in solution velocities of 20–80 m s^−1^. The solutions were prepared by diluting alanine in water, adjusting solution pH where necessary with concentrated HCl or solid NaOH, and finally diluting the solution to reach a final concentration of 1 M alanine. The pH 6 solutions represent as-prepared solutions, without the addition of either NaOH or HCl. The solutions were injected horizontally and frozen out after the interaction region *via* contact with a liquid-nitrogen-cooled cold trap. All PES experiments were performed at the P04 soft X-ray beamline of the PETRA III storage ring at DESY in Hamburg, Germany. Critically, the P04 beamline is equipped with an APPLE-II undulator, enabling experiments with circularly polarized light within the experimentally relevant photon-energy range of 290–320 eV.^71^ Although not available during our early measurements, recent advancements at the P04 beamline now enable the estimation of the degree of circular polarization for a given set of undulator conditions. Retroactive estimates revealed an expected circular polarization of >99% for all experimental conditions employed in this study. d-, l-, and dl-alanine (>98% purity) were purchased from Sigma-Aldrich and Carl Roth and used as received. Enantiomeric purity was confirmed *via* intermittent testing using ECD (see Fig. S2†). For additional experimental details regarding our instrumentation or LJ-PES more generally, we direct interested readers to our recent technical publication.^29^ All data reduction was performed using the Igor Pro analysis software (Wavemetrics, version 9).

## Data availability

The data of relevance to this study may be accessed at the following DOI: 10.5281/zenodo.13902253.

## Author contributions

B. W. conceived the experiments. D. S., S. T., F. T., U. H., I. W., L. N., G. M., I. P., and B. W. planned the experiments. D. S., F. T., U. H., M. P., B. C., S. M., I. W., and B. W. prepared and carried out the experiments. D. S., S. T., U. H., and I. P. developed the methodology used to analyze the data. D. S., and S. T. formally analyzed the data. D. S., S. T., I. P., and B. W. wrote the manuscript with feedback from all authors.

## Conflicts of interest

There are no conflicts of interest to declare.

## Supplementary Material

SC-OLF-D5SC00167F-s001
