# Supplementary material for: Photoelectron circular dichroism of aqueous-phase alanine
Source: Chem Sci. 2025 Apr 7;16(20):8637–47. doi: 10.1039/d5sc00167f (PMC12004263; doi:10.1039/d5sc00167f)
Supplement: SC-016-D5SC00167F-s001 [file SC-016-D5SC00167F-s001.pdf]

Supplementary Information (SI) for Chemical Science.
This journal is © The Royal Society of Chemistry 2025

Supplementary Information: Photoelectron Circular Dichroism of Aqueous-Phase Alanine

Dominik Stemer,*,† Stephan Thürmer,*,‡ Florian Trinter,† Uwe Hergenhahn,† Michele Pugini,† Bruno Credidio,† Sebastian Malerz,¶ Iain Wilkinson,§ Laurent Nahon,‖ Gerard Meijer,† Ivan Powis,⊥ and Bernd Winter*,†

†*Fritz-Haber-Institut der Max-Planck-Gesellschaft, Berlin 14195, Germany.*

‡*Department of Chemistry, Kyoto University, Kyoto 606-8502, Japan.*

¶*Department of Optics and Beamlines, Helmholtz-Zentrum Berlin für Materialien und Energie, Berlin 14109, Germany.*

§*Institute for Electronic Structure Dynamics, Helmholtz-Zentrum Berlin für Materialien und Energie, Berlin 14109, Germany.*

‖*Synchrotron SOLEIL, St. Aubin 91190, France.*

⊥*School of Chemistry, The University of Nottingham, Nottingham NG7 2RD, UK.*

E-mail: dstemer@fhi-berlin.mpg.de; thuermer@kuchem.kyoto-u.ac.jp; winter@fhi-berlin.mpg.de

Background Subtraction and Data Analysis

We attempted many approaches to background subtraction, including the use of an experimental background measured for neat water solutions without alanine under the same experimental conditions for which we measured the corresponding alanine spectra. However, we found that such experimentally measured background spectra provided an imperfect fit to the data, and we were unable to sufficiently improve the fit through simple scaling of the spectra. In general, the measurement of an appropriate background spectrum for low-KE PES of condensed-phase targets is not straightforward. Although the simple measurement of a reference sample (e.g. pure water) would seem to provide a reasonable background, the

measured low-energy electron background spectrum of any sample will naturally strongly depend on the integral ionization cross section of all species contained in the sample, their surface propensities, and the specific electron mean free path within that sample. These effects combined to make the measurement of an appropriate experimental background for kinetic energies below 15–20 eV very challenging. We also attempted to measure experimental background spectra by keeping the solution unchanged, and instead increasing the photon energy sufficiently such that the alanine C 1s features were pushed to higher kinetic energy and out of the measurement range. This approach also proved unsuitable, as all of the previously listed factors depend critically on photon energy. Ultimately, we chose to fit the background using a variety of functions and determined that polynomials and sums of polynomials and exponentials constituted the most effective and reproducible background function for these spectra.

Data were analyzed pairwise and for each pair of intensity-corrected spectra, we manually defined a region of interest containing all three of the C 1s peaks and performed a fit to the background with this region masked. We were careful to avoid overfitting, and only accepted a fitted background if small changes to the masked region did not greatly influence the resulting peak shapes and intensities. We then calculated the point-wise difference-over-sum for the + and − data as appears in Eq. 3. This was repeated for the data prior to background subtraction; these data served as a guide during background subtraction and are presented below (Figs. S5, S6, S9, S10, S13, and S14). We identified the peak positions in the background-subtracted data by locating maxima in the second derivatives of the spectra. For each peak, we defined a kinetic-energy window centered at the peak position with a width defined by the full width at half maximum of the peak. Minor differences in peak positions due to offsets in photon energy between left- and right-handed CPL beamline settings were usually less than 5–10 meV and were corrected manually prior to determination of b_1^{+1}. We then calculated the mean and standard deviation for the values of the asymmetry arising from the data within these energy windows. These asymmetries were subsequently multiplied by a geometric factor taking into account the measurement angle of 50°, resulting in a single value of b_1^{+1} per peak based on Eq. 2. This process is illustrated in Fig. S1

A single datapoint, as presented in Fig. S5, thus constitutes the comparison between a pair of spectra measured with left- and right-handed CPL. Each spectrum used for such analysis is in general taken as an average of 10 acquisitions. The time investment for a single data point is approximately one hour of measurement time.

Electronic Circular Dichroism

Absorption-based electronic circular-dichroism measurements were performed using a commercial Chirascan 100 CD spectrophotometer (Applied Photophysics). Dilute (50 mM) aqueous solutions of D-, L-, and DL-alanine were prepared using ultrapure water without pH adjustment, and measured in a quartz cuvette with a 1 mm path length. Electronic circular dichroism spectra were recorded in the wavelength range of 195–250 nm with a step size of 1 nm. For each sample, we measured 3 spectra. The averaged results are presented in Fig. S2

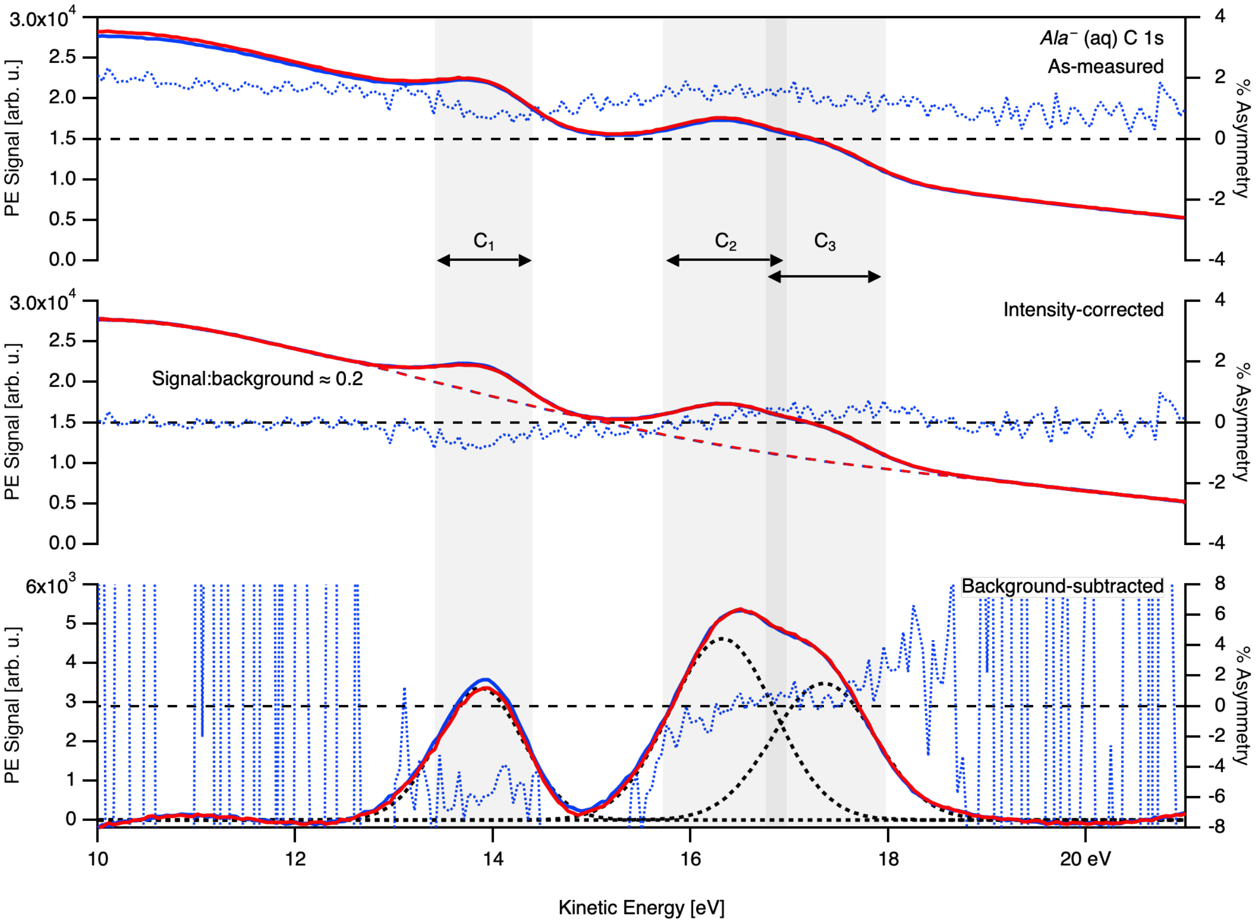

Figure S1: Example of data analysis workflow for a sample pair of spectra measured for Ala^{-} with hν=307 eV photons. Top: As-measured spectra measured with left- and right-handed circularly polarized light (blue and red solid lines, respectively), with calculated asymmetry (dotted blue line). Middle: Intensity-corrected spectra and associated background (dashed red and blue lines), with calculated asymmetry. Bottom: Background-subtracted spectra, with associated C1, C2, and C3 peak fits and calculated asymmetry. The gray areas denote the kinetic-energy range within which the asymmetry values are averaged to produce a single value of b_1^{+1}. Note that the magnitude of the asymmetry calculated after background subtraction is significantly larger than that calculated prior to background subtraction, reflecting the low signal-to-background ratio of the as-measured data.

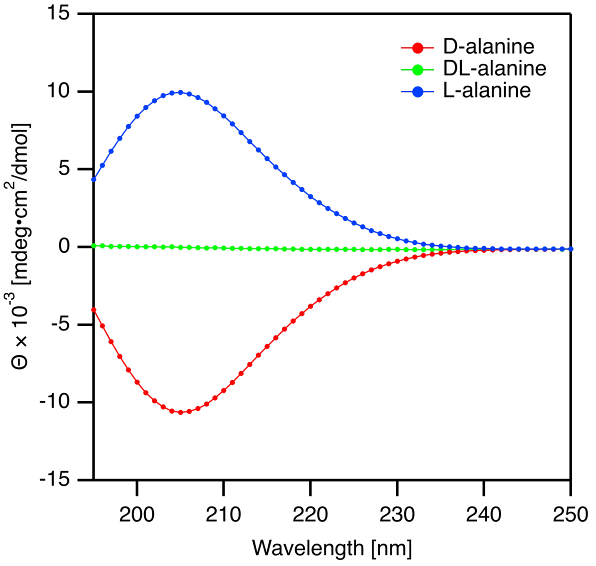

Figure S2: Electronic circular dichroism absorption spectra for 50 mM aqueous solutions of D-, L-, and DL-alanine (red, blue, and green lines, respectively). The data demonstrate clear optical activity only for the enantiopure samples. All data are plotted in units of molar ellipticity.

Calculation of Alanine b_2^0 Photoionization Parameter

Calculations to predict the b_2^0 angular distribution parameter for C 1s photoemission from alanine were made using the CMS-Xα method as fully described and applied previously by Tia et al .[1] Those calculations examined the isolated (gas-phase) neutral molecule's valence photoionization but were here extended to treat core-level photoemission from the three carbon sites. Three different conformers of alanine were modeled, believed to be the most stable configurations of the gas-phase molecule. Conformers 1, 2, and 3 correspond to those descriptions used in reference 1. In the kinetic-energy range investigated, the β parameter for core-level photoionization was found to display little conformer dependence.

Photoelectron Circular Dichroism Data for all Carbon Groups

Although the primary focus of our study was on photoelectron circular dichroism in photoionization of alanine's COOH/COO^- group, we also collected data for alanine's other carbon centers. Here, we include data corresponding to photoionization of alanine's chiral center (C_2) and methyl group (C_3), as well as values for b_1^+ based only on the measured data prior to background subtraction.

Error Propagation for Binned Datasets

For clarity, we have displayed the data in the main text following binning of data points using a 250 meV kinetic-energy window. The shaded regions in these plots represent the combined

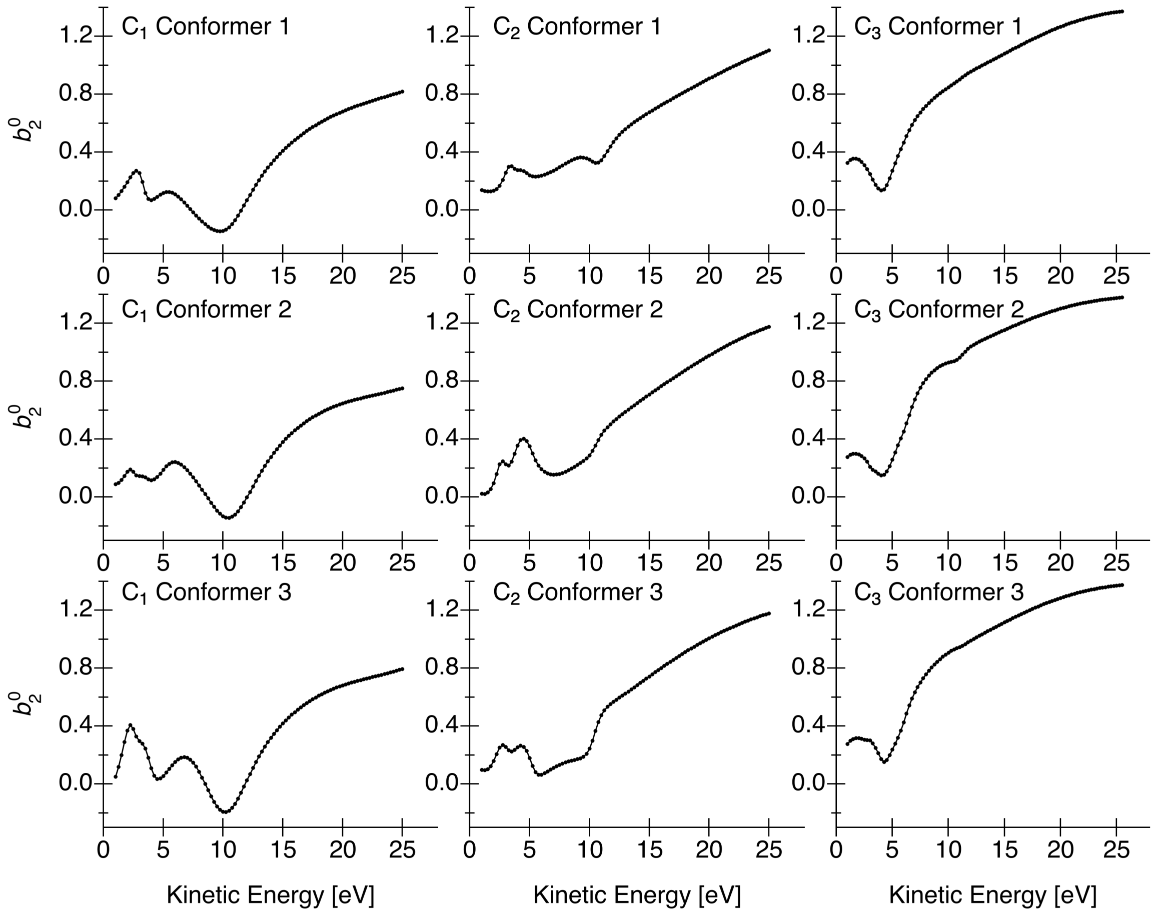

Figure S3: Calculated values of b_2^0 for 1s photoionization of alanine's three carbon groups as a function of photoelectron kinetic energy. C_1, C_2, and C_3 correspond to alanine's carboxylic group, chiral center, and methyl group, respectively, as described in the main text. Conformers 1, 2, and 3 are the same as those discussed in reference 1.

error of the individual data points as well as the spread of the data points binned. The error of each data point was calculated as the standard deviation of the percent difference across the full width at half maximum of the PE feature (see Fig. 3). When n data points were binned together, the error was calculated as:

$$Error = \sqrt{\frac{\sum_{i=1}^{n} \sigma_i^2}{n^2} + \left(\frac{\sigma_{bin}}{\sqrt{n}}\right)^2}, \quad (1)$$

with σ_i being the error of a given point, and σ_{bin} the standard deviation of the n values of b_1^{+1} in a given bin. The relation propagates the individual error of each data point and the standard error of the mean of all data points within a binning window.

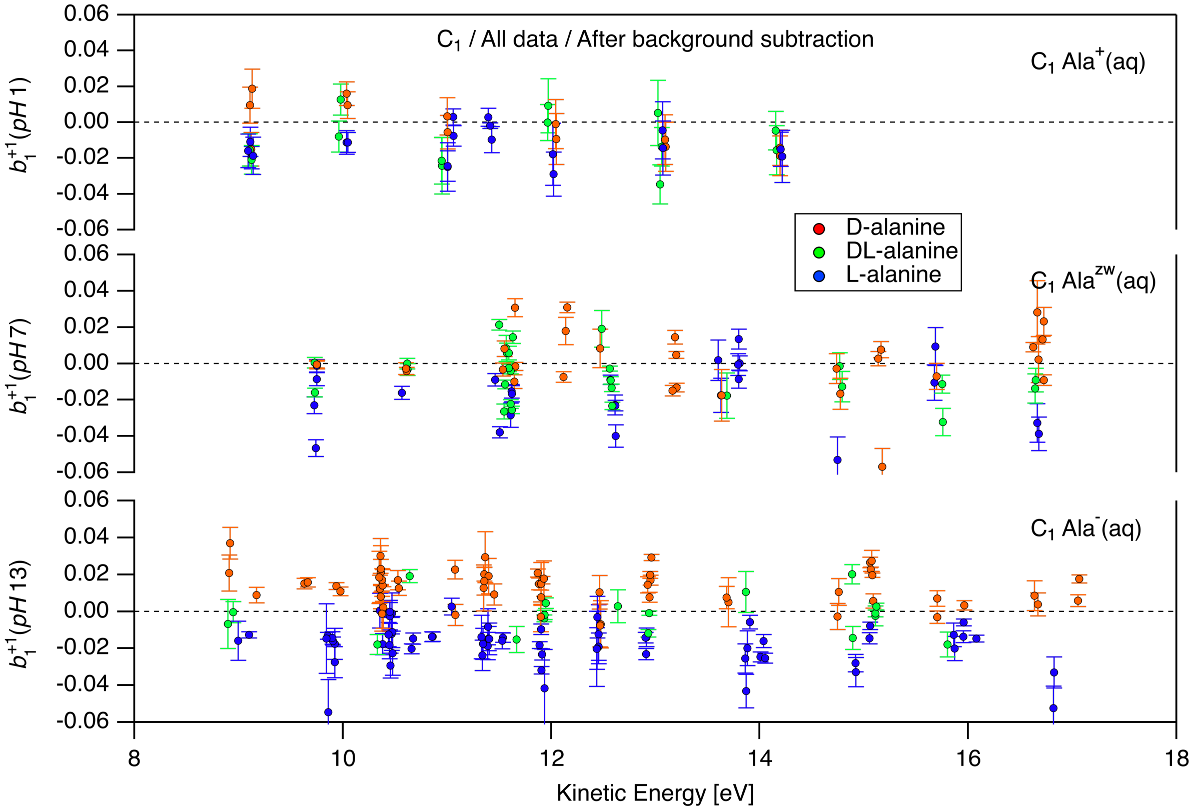

Figure S4: Values of the b_1^{+1} photoionization parameter obtained for C 1s measurements of aqueous solutions of D-, L-, and DL-alanine (red, blue, and green points, respectively) at pH 1, 7, and 13 (top, middle, and bottom; corresponding to the cationic, zwitterionic, and anionic form of the molecule, respectively). All b_1^{+1} values shown correspond to photoionization of the C_1 carboxylic acid group.

References

(1) Tia, M.; Cunha De Miranda, B.; Daly, S.; Gaie-Levrel, F.; Garcia, G. A.; Nahon, L.; Powis, I. VUV photodynamics and chiral asymmetry in the photoionization of gas phase

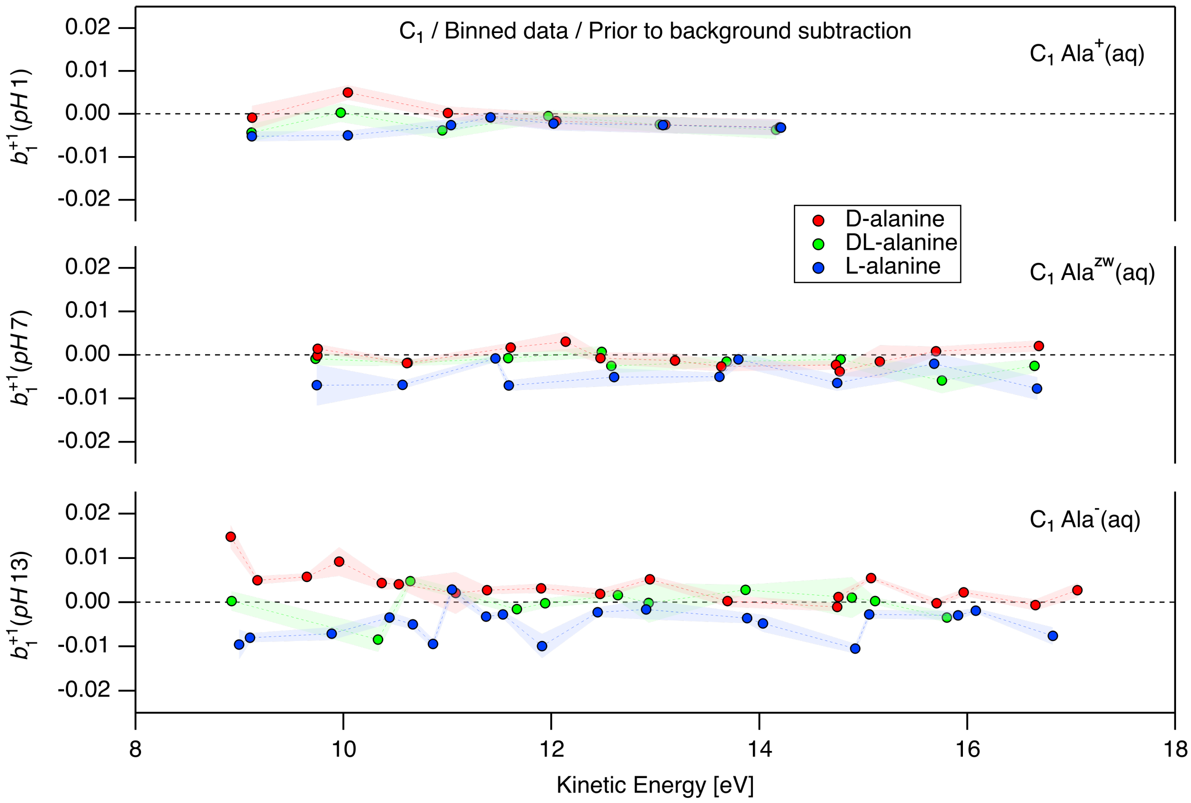

Figure S5: Values of the b_1^{+1} photoionization parameter obtained for C 1s measurements of aqueous solutions of D-, L-, and DL-alanine (red, blue, and green points, respectively) at pH 1, 7, and 13 (top, middle, and bottom; corresponding to the cationic, zwitterionic, and anionic form of the molecule, respectively). All b_1^{+1} values shown correspond to photoionization of the C_1 carboxylic acid group prior to background subtraction. The data is displayed with a kinetic-energy binning of 250 meV.

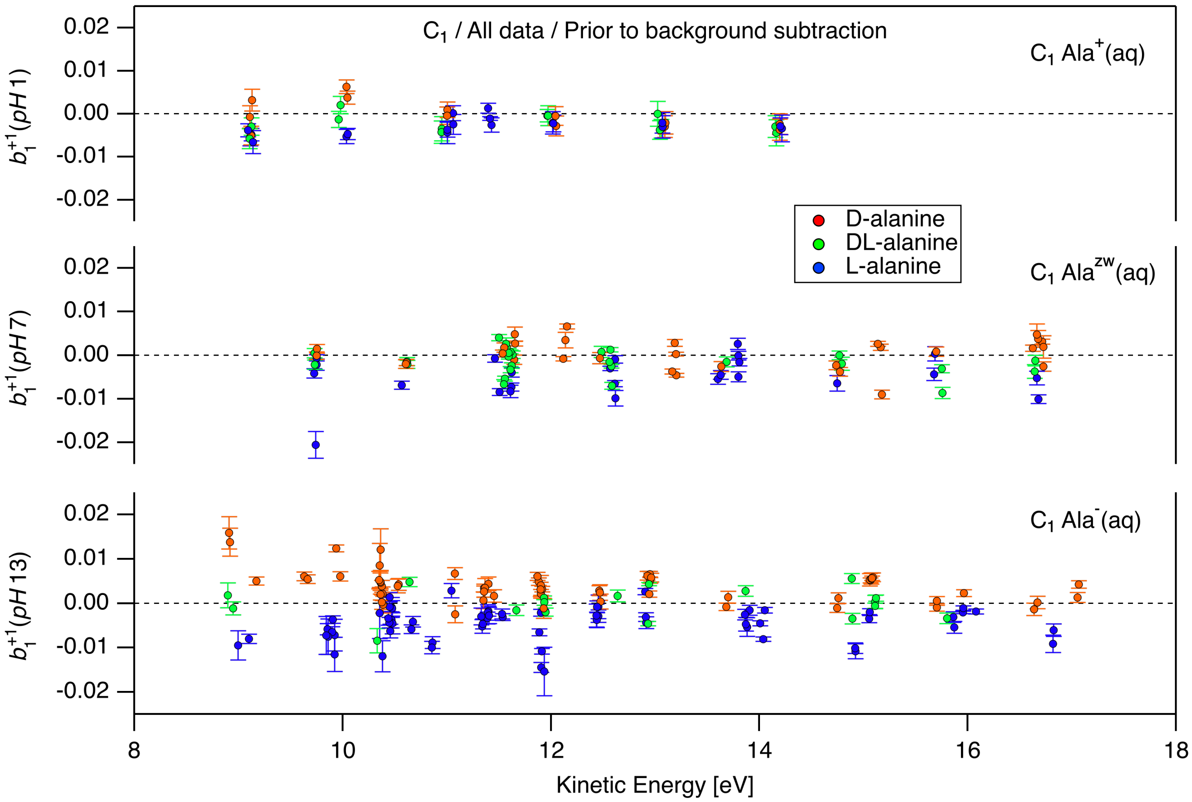

Figure S6: Values of the b_1^{+1} photoionization parameter obtained for C 1s measurements of aqueous solutions of D-, L-, and DL-alanine (red, blue, and green points, respectively) at pH 1, 7, and 13 (top, middle, and bottom; corresponding to the cationic, zwitterionic, and anionic form of the molecule, respectively). All b_1^{+1} values shown correspond to photoionization of the C_1 carboxylic acid group prior to background subtraction.

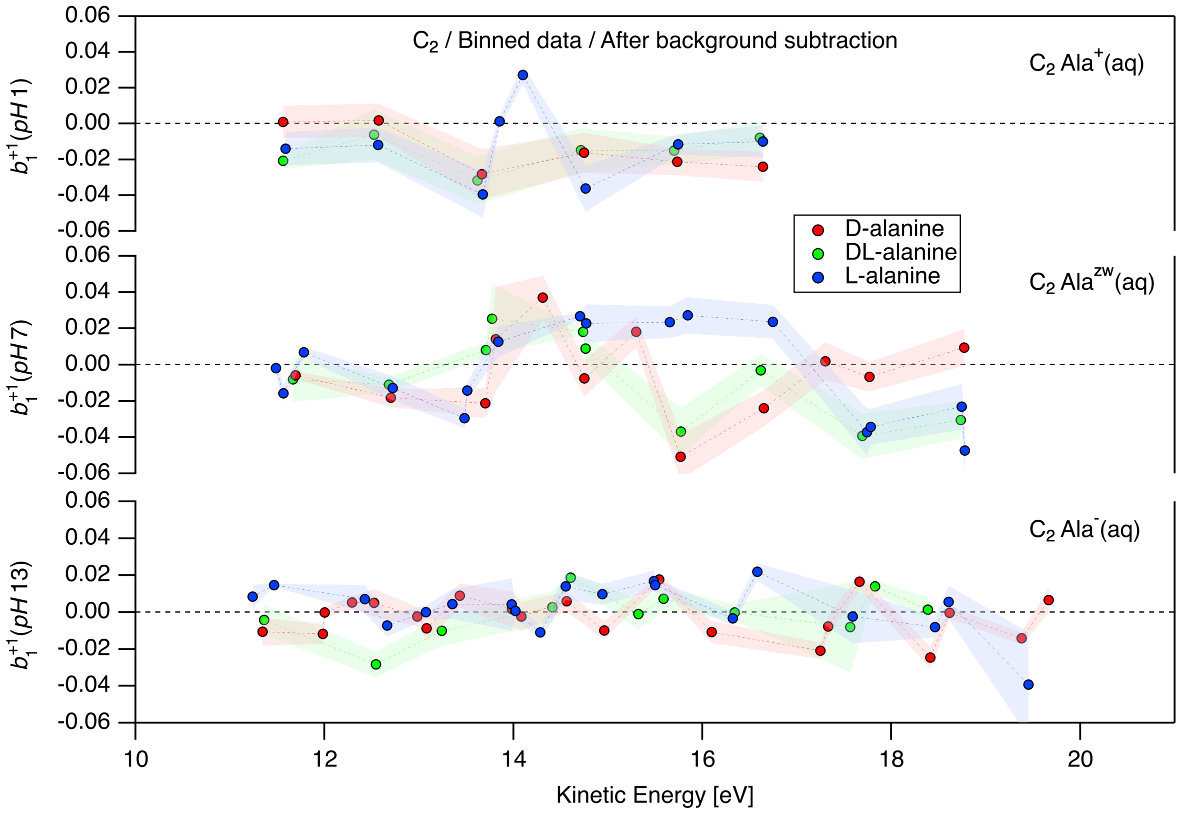

Figure S7: Values of the b_1^{+1} photoionization parameter obtained for C 1s measurements of aqueous solutions of D-, L-, and DL-alanine (red, blue, and green points, respectively) at pH 1, 7, and 13 (top, middle, and bottom; corresponding to the cationic, zwitterionic, and anionic form of the molecule, respectively). All b_1^{+1} values shown correspond to photoionization of the C_2 chiral center. The data is displayed with a kinetic-energy binning of 250 meV.

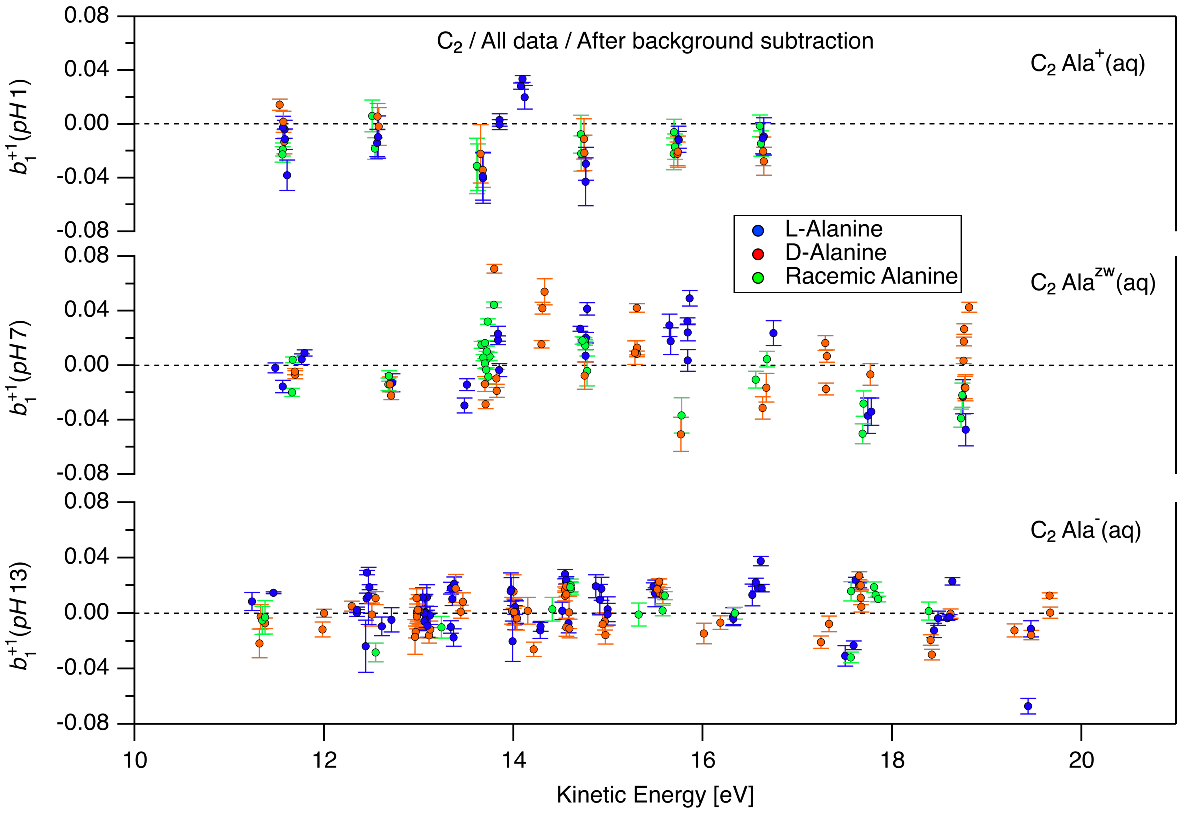

Figure S8: Values of the b_1^{+1} photoionization parameter obtained for C 1s measurements of aqueous solutions of D-, L-, and DL-alanine (red, blue, and green points, respectively) at pH 1, 7, and 13 (top, middle, and bottom; corresponding to the cationic, zwitterionic, and anionic form of the molecule, respectively). All b_1^{+1} values shown correspond to photoionization of the C_2 chiral center.

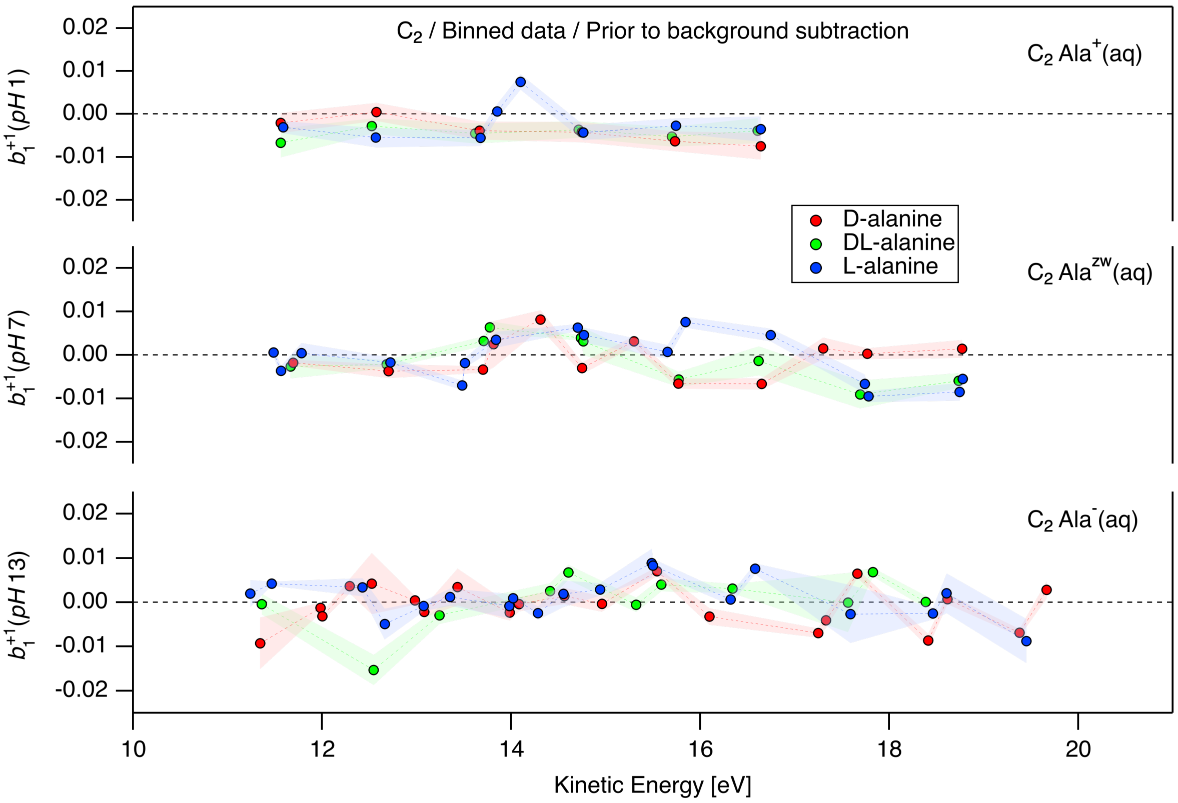

Figure S9: Values of the b_1^{+1} photoionization parameter obtained for C 1s measurements of aqueous solutions of D-, L-, and DL-alanine (red, blue, and green points, respectively) at pH 1, 7, and 13 (top, middle, and bottom; corresponding to the cationic, zwitterionic, and anionic form of the molecule, respectively). All b_1^{+1} values shown correspond to photoionization of the C_2 chiral center prior to background subtraction. The data is displayed with a kinetic-energy binning of 250 meV.

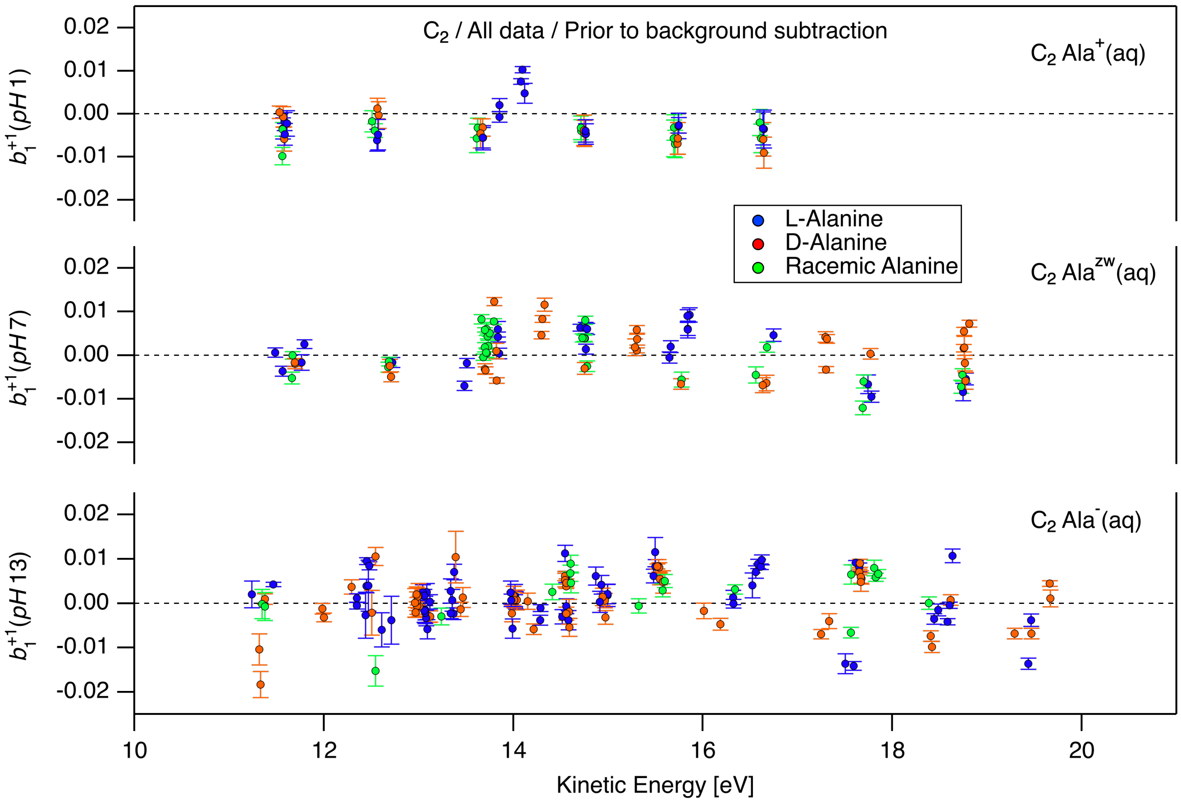

Figure S10: Values of the b_1^{+1} photoionization parameter obtained for C 1s measurements of aqueous solutions of D-, L-, and DL-alanine (red, blue, and green points, respectively) at pH 1, 7, and 13 (top, middle, and bottom; corresponding to the cationic, zwitterionic, and anionic form of the molecule, respectively). All b_1^{+1} values shown correspond to photoionization of the C_2 chiral center prior to background subtraction.

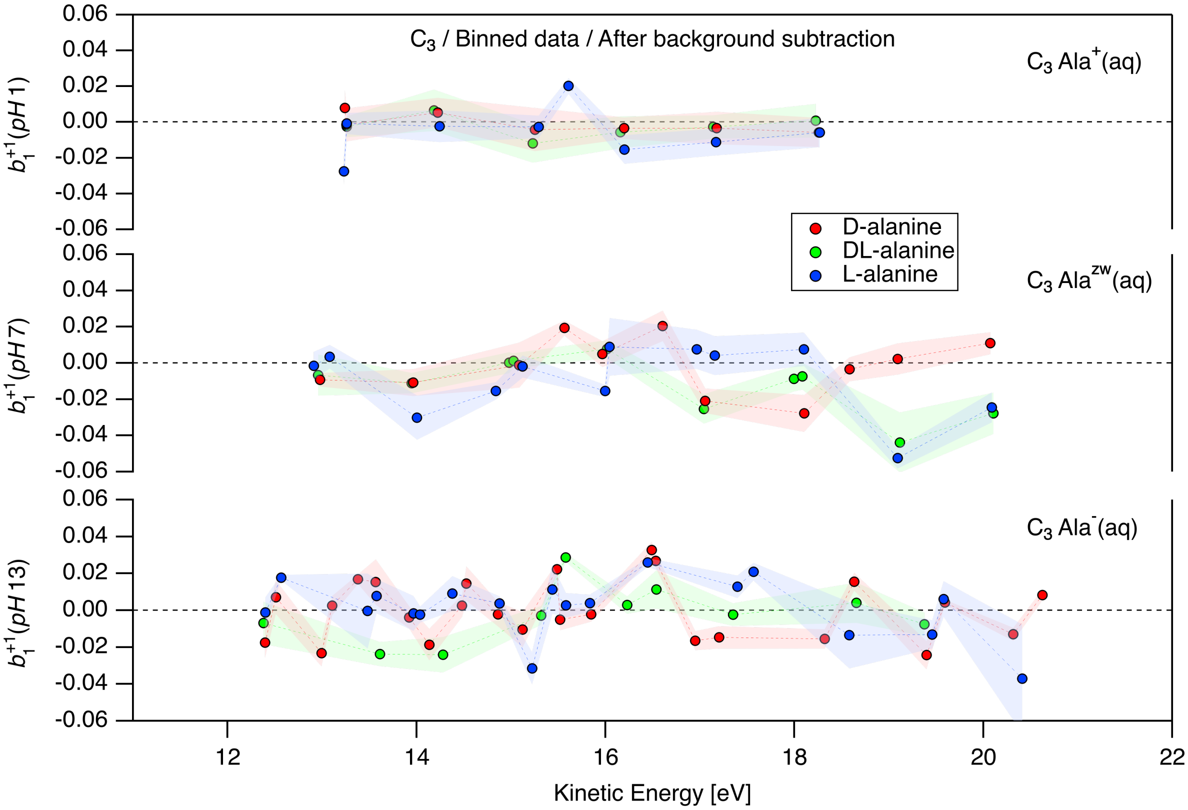

Figure S11: Values of the b_1^{+1} photoionization parameter obtained for C 1s measurements of aqueous solutions of D-, L-, and DL-alanine (red, blue, and green points, respectively) at pH 1, 7, and 13 (top, middle, and bottom; corresponding to the cationic, zwitterionic, and anionic form of the molecule, respectively). All b_1^{+1} values shown correspond to photoionization of the C_3 methyl group. The data is displayed with a kinetic-energy binning of 250 meV.

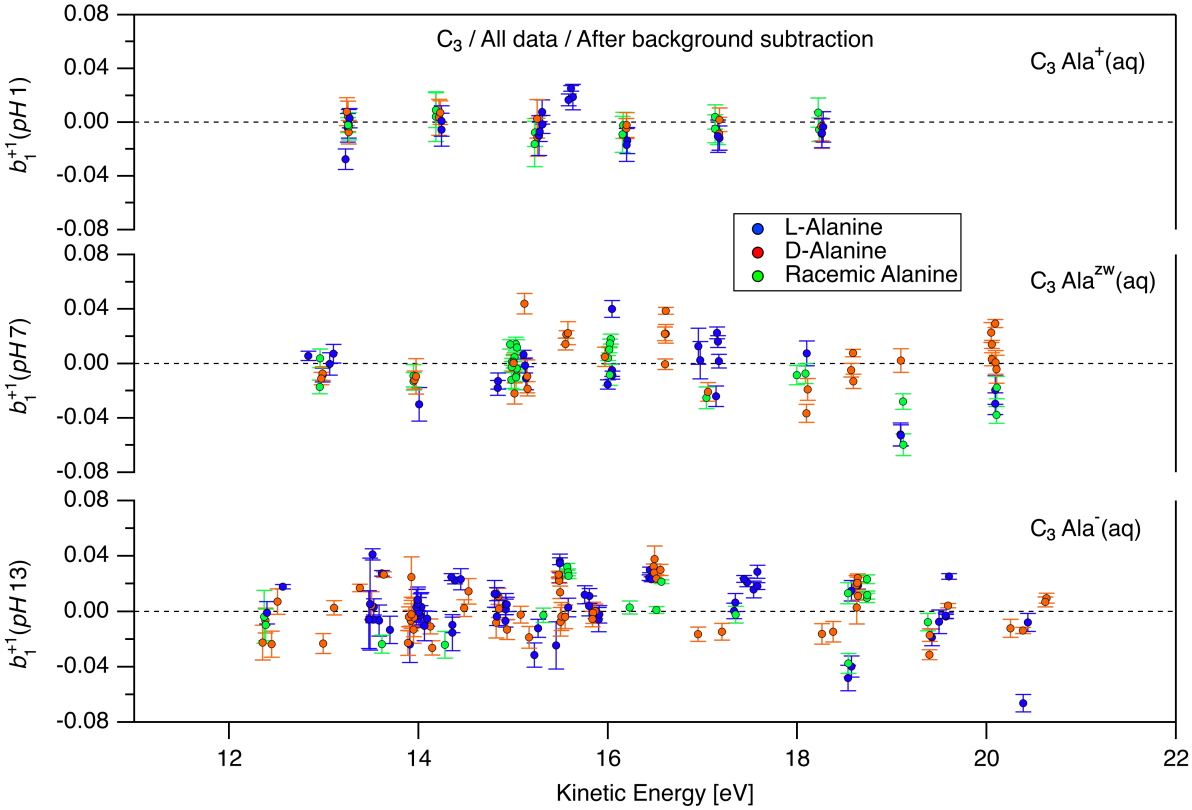

Figure S12: Values of the b_1^{+1} photoionization parameter obtained for C 1s measurements of aqueous solutions of D-, L-, and DL-alanine (red, blue, and green points, respectively) at pH 1, 7, and 13 (top, middle, and bottom; corresponding to the cationic, zwitterionic, and anionic form of the molecule, respectively). All b_1^{+1} values shown correspond to photoionization of the C_3 methyl group.

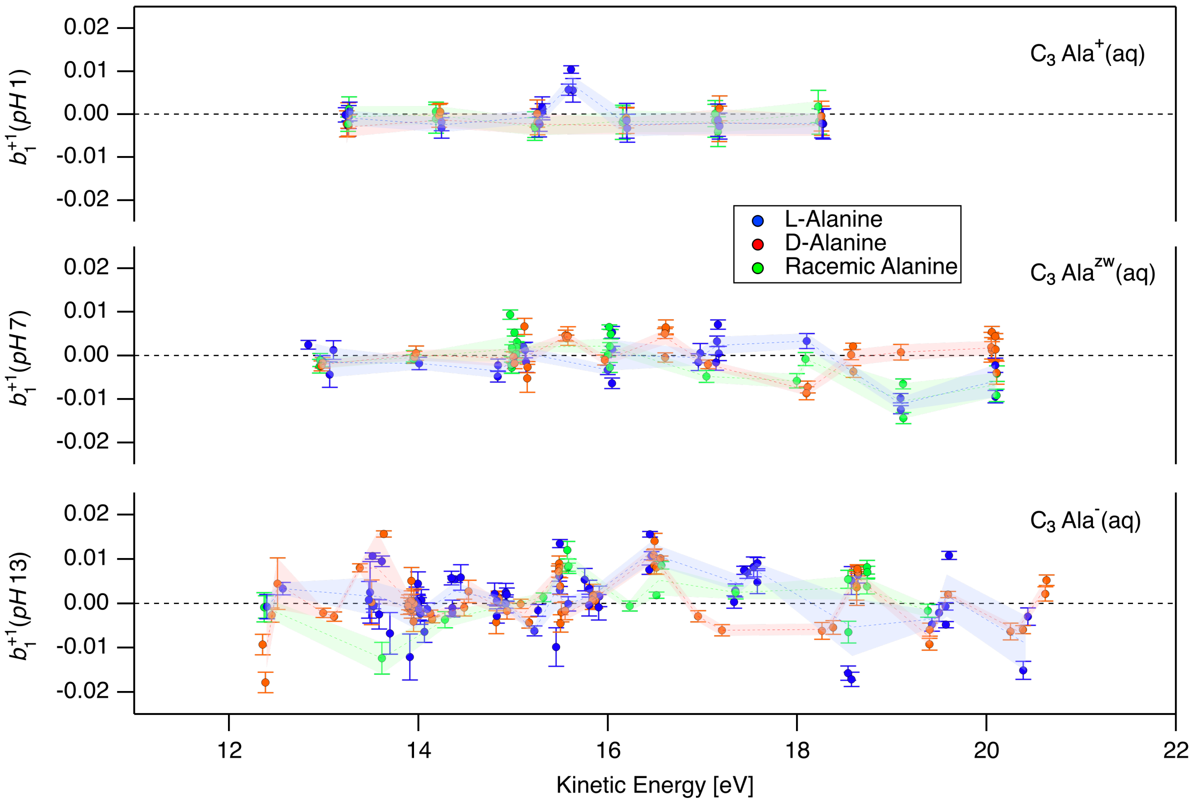

Figure S13: Values of the b_1^{+1} photoionization parameter obtained for C 1s measurements of aqueous solutions of D-, L-, and DL-alanine (red, blue, and green points, respectively) at pH 1, 7, and 13 (top, middle, and bottom; corresponding to the cationic, zwitterionic, and anionic form of the molecule, respectively). All b_1^{+1} values shown correspond to photoionization of the C_3 methyl group prior to background subtraction. The data is displayed with a kinetic-energy binning of 250 meV.

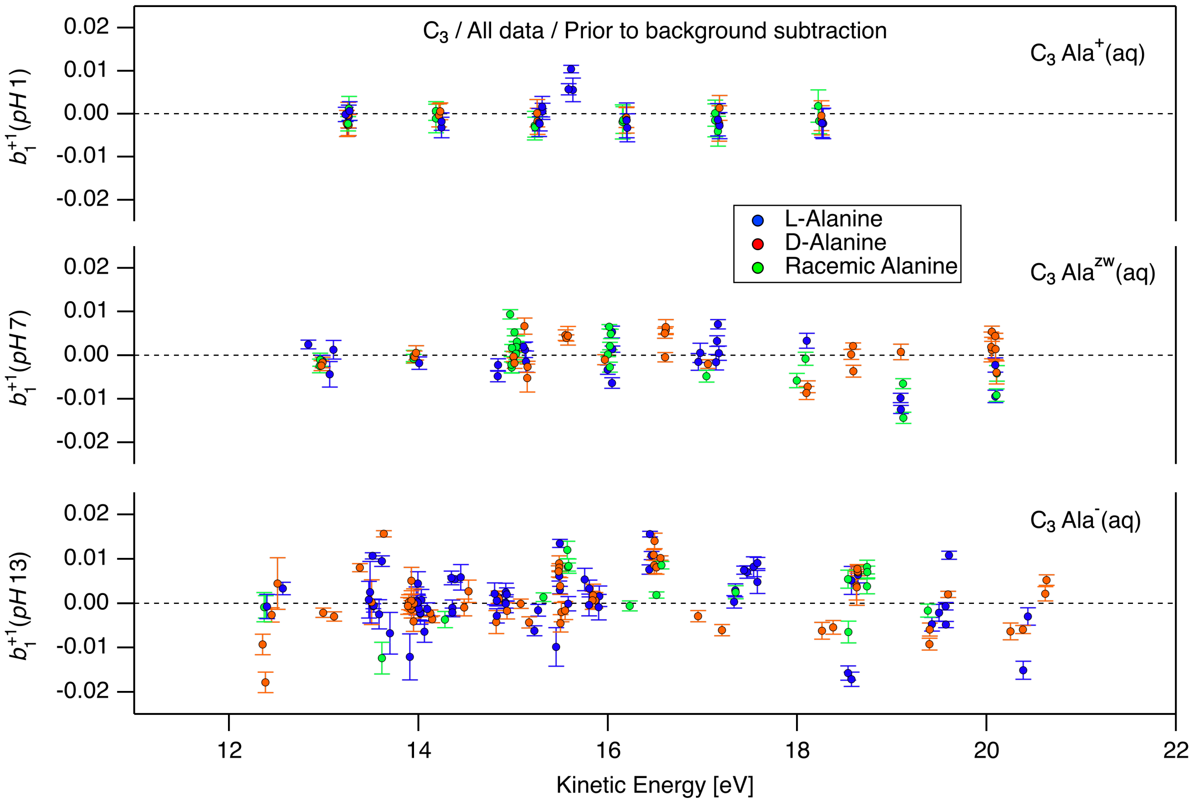

Figure S14: Values of the b_1^{+1} photoionization parameter obtained for C 1s measurements of aqueous solutions of D-, L-, and DL-alanine (red, blue, and green points, respectively) at pH 1, 7, and 13 (top, middle, and bottom; corresponding to the cationic, zwitterionic, and anionic form of the molecule, respectively). All b_1^{+1} values shown correspond to photoionization of the C_3 methyl group prior to background subtraction.

alanine enantiomers. *J. Phys. Chem. A* **2014**, *118*, 2765–2779.
